# The role of glucose metabolic reprogramming in myocarditis and advances in therapeutic strategies

**DOI:** 10.3389/fcvm.2026.1781627

**Published:** 2026-03-17

**Authors:** Xingchen Liu, Bo Han

**Affiliations:** Shandong Provincial Hospital Affiliated to Shandong First Medical University, Jinan, China

**Keywords:** glucose, glycolysis, immunometabolism, metabolic, myocarditis, regulatory mechanisms, reprogramming, therapeutic targets

## Abstract

Myocarditis is a heterogeneous inflammatory heart disease most commonly triggered by viral infections, such as Coxsackievirus B3, and may progress to dilated cardiomyopathy and heart failure. Growing evidence highlights the pivotal role of glucose metabolic reprogramming in cardiomyocytes and infiltrating immune cells during the initiation and progression of myocarditis. Under physiological conditions, the adult heart primarily relies on fatty acid β-oxidation for energy production, with glucose oxidation serving a supplementary role. In contrast, myocarditis is characterized by a metabolic shift from oxidative phosphorylation toward enhanced aerobic glycolysis, known as the Warburg effect. This shift results in reduced ATP efficiency, lactate accumulation, excessive reactive oxygen species production, and amplification of inflammatory responses, thereby establishing a self-sustaining immunometabolic vicious cycle. This review summarizes glucose metabolism in the normal heart and highlights the features and regulatory mechanisms of glucose metabolic reprogramming in myocarditis, including the hypoxia-inducible factor-1α/mammalian target of rapamycin axis, nuclear factor erythroid 2-related factor 2-mediated pentose phosphate pathway, immune-responsive gene 1/itaconate axis, and phosphoglycerate kinase 1. Emerging therapeutic strategies targeting glucose metabolism are discussed, as well as current challenges in clinical translation. Advances in multiomics technologies may facilitate the development of precise metabolic interventions for myocarditis.

## 1 Introduction

Myocarditis is a heterogeneous disease characterized by myocardial inflammation and cardiomyocyte injury (1), with etiologies encompassing infectious agents, autoimmune abnormalities, and toxic insults (2, 3). Its clinical presentation is highly variable, ranging from mild and self-limiting disease to fulminant heart failure, malignant arrhythmias, and sudden cardiac death. Among the different etiological subtypes, viral myocarditis is the most prevalent, with Coxsackievirus B3 (CVB3) (4), Parvovirus B19 (5), and human herpesvirus 6 recognized as the principal causative pathogens (6). Although advances in molecular diagnostic techniques have improved pathogen detection, current therapeutic approaches for myocarditis remain largely supportive, and effective targeted interventions are still lacking.

The natural course of myocarditis exhibits a distinct stage-dependent progression (7). The acute phase is typically dominated by viral replication or initial immune activation, followed by an immune-mediated injury phase (8). In a subset of patients, persistent inflammation leads to progressive structural and functional cardiac remodeling, ultimately resulting in inflammatory cardiomyopathy or dilated cardiomyopathy (DCM) (9). Accumulating evidence indicates that disease outcomes are not determined solely by the pathogenic agent but are closely associated with the magnitude, duration, and regulatory efficiency of the host immune response (10). Immune dysregulation is therefore considered a major determinant of sustained myocardial injury and irreversible deterioration of cardiac function (11).

Within this pathological context, alterations in cardiac energy metabolism have attracted increasing attention (12). Under physiological conditions, the adult heart relies predominantly on mitochondrial oxidative phosphorylation to meet its energy demands, with fatty acid β-oxidation accounting for approximately 70%–90% of ATP production, while glucose oxidation provides supplementary support (13). This metabolic configuration is well suited to sustain the long-term, high-energy requirements of myocardial contraction. However, during myocarditis, local inflammation, impaired oxygen delivery, and mitochondrial structural and functional damage are commonly observed, leading to profound alterations in cardiomyocyte energy metabolism (14–16).

Experimental studies have consistently demonstrated that cardiomyocytes in myocarditis exhibit a metabolic shift from oxidative phosphorylation toward increased reliance on glycolysis, even under relatively oxygen-replete conditions (17). This phenotype closely resembles aerobic glycolysis, also known as the Warburg effect. Although enhanced glycolysis may transiently support cellular energy demands, it is markedly less efficient in ATP generation and is accompanied by lactate accumulation, increased production of reactive oxygen species (ROS), and activation of multiple metabolic bypass pathways. tripartite motif-containing 29 (TRIM29), as a multifunctional E3 ubiquitin ligase, has not been directly studied in myocarditis. However, its key roles in regulating core pathways, such as the type I interferon antiviral response and NLRP3 inflammasome activation, strongly suggest that it may serve as a critical molecular node linking viral infection, excessive immune response, and myocardial injury (18–20). These metabolic disturbances are closely associated with cardiomyocyte dysfunction, cell death, and sustained inflammatory responses (21–23).

In addition to cardiomyocytes, infiltrating immune cells within the myocardium undergo substantial metabolic remodeling. Pro-inflammatory macrophages and effector T cells predominantly rely on glycolysis to sustain their inflammatory functions, whereas anti-inflammatory macrophages and regulatory T cells preferentially utilize oxidative phosphorylation and fatty acid oxidation (24). In the context of myocarditis, the metabolic state of immune cells directly influences the magnitude and persistence of inflammation and, consequently, modulates myocardial injury and repair (25). This tight coupling between metabolic programming and immune function positions glucose metabolic reprogramming as a critical nexus linking inflammation to myocardial pathology.

With advances in immunometabolism research, multiple glucose metabolism-related signaling pathways have been identified as contributors to the pathogenesis of myocarditis, including hypoxia-inducible factor-1α (HIF-1α) (26), mammalian target of rapamycin (mTOR) (27), nuclear factor erythroid 2-related factor 2 (NRF2) (28), the immune-responsive gene 1 (IRG1)/itaconate pathway, and phosphoglycerate kinase 1 (PGK1) (17, 29). These regulators play central roles in controlling glycolytic flux, redox homeostasis, and immune cell differentiation, and they exhibit clear pathological relevance in experimental models of myocarditis (30, 31).

In recent years, therapeutic strategies targeting glucose metabolic reprogramming have demonstrated potential benefits in multiple experimental models of myocarditis (32). Interventions aimed at inhibiting glycolysis, blocking key metabolic enzymes, or activating protective metabolic pathways have been shown to attenuate myocardial inflammation and improve cardiac function to varying degrees (33). Nevertheless, metabolic heterogeneity among different cell types, disease stages, and experimental models highlights the need for more refined mechanistic understanding and careful risk assessment before clinical translation (34, 35).

Based on these advances, this review systematically summarizes the characteristics and regulatory mechanisms of glucose metabolic reprogramming in myocarditis, with particular emphasis on the interplay between metabolic alterations in cardiomyocytes and immune cells (36). Furthermore, we discuss current progress and challenges in targeting metabolic pathways as therapeutic strategies, aiming to provide a theoretical foundation for the development of precise metabolic interventions in myocarditis.

## 2 Pathophysiological basis of myocarditis

The true incidence of myocarditis in the general population has long been considered underestimated. Epidemiological studies suggest that the annual incidence of approximately 10–22 per 100,000 individuals, with a markedly higher prevalence observed in children and young adults (36). Among cases of sudden cardiac death in individuals younger than 35 years, myocarditis is regarded as one of the leading causes, second only to inherited arrhythmogenic disorders (37). Analyses of hospitalized patient cohorts indicate that myocarditis accounts for approximately 5%–15% of non-ischemic cardiomyopathies, and a substantial proportion of patients continue to exhibit persistent cardiac dysfunction following the acute phase (38).

Sex and age are important determinants influencing both the incidence and clinical outcome of myocarditis (38). The disease occurs significantly more frequently in men than in women, with an approximate male-to-female ratio of 2:1, a difference that is thought to be related to sex hormone-mediated modulation of immune responses and viral clearance capacity (38). Viral myocarditis is more commonly observed in children and adolescents, whereas middle-aged and elderly patients more often present with autoimmune predisposition or metabolic abnormalities. In fulminant myocarditis, although short-term mortality is relatively high, survivors may achieve favorable long-term outcomes with timely supportive treatment. In contrast, subacute myocarditis and chronic myocarditis are more likely to progress to DCM (39, 40).

Myocarditis is most commonly initiated by the introduction of pathogenic viral strains, such as Coxsackieviruses, or by reactivation of latent pathogens, including Parvovirus B19 (41, 42).Viruses can replicate within permissive tissues of susceptible hosts and subsequently disseminate to the myocardium or vascular structures via hematogenous or lymphatic routes (43). The balance of the host immune response is a major determinant of clinical outcome. On the one hand, immune activation is required to eliminate infected cells and control viral replication (43). On the other hand, failure to appropriately regulate and terminate the inflammatory response may result in excessive tissue injury and direct organ dysfunction (44). Host antiviral defense relies on both innate and adaptive immunity, with multiple immune components contributing to the pathogenesis of myocarditis across acute infection, subacute immune responses, and maladaptive cardiac remodeling (45).

Innate immune responses are initiated through pattern recognition receptors expressed on innate immune cells. These receptors recognize pathogen-associated molecular patterns and damage-associated molecular patterns (DAMPs) released from injured cells, thereby promoting the release of cytokines, chemokines, and alarmins (46). Subsequently, adaptive immune responses lead to the recruitment of inflammatory cells to the heart, including mast cells, neutrophils, dendritic cells, monocytes, and macrophages (47). Notably, monocytes and macrophages represent the predominant immune cell populations in myocarditis. Upon arrival at sites of injury, monocytes further differentiate into macrophages with distinct functional phenotypes in response to cytokine stimulation, thereby participating in both acute and chronic inflammatory processes within the myocardium (47). If inflammatory activation persists, progressive cardiac remodeling may ensue, ultimately resulting in structural and functional alterations characteristic of DCM (48).

Thus, inflammation constitutes the central pathological axis throughout the initiation, progression, and outcome of myocarditis. It not only serves as the direct effector mechanism of cardiomyocyte injury but also represents the key driver linking acute myocardial damage to chronic cardiac remodeling (49). Under physiological conditions, energy (ATP) production in healthy cardiomyocytes is highly dependent on mitochondrial oxidative phosphorylation, utilizing substrates such as fatty acids and glucose through an efficient electron transport chain. Fatty acid β-oxidation accounts for approximately 70%–90% of ATP generation, whereas glucose oxidation provides a supplementary contribution of 10%–30%, representing a highly efficient, oxygen-dependent metabolic program (50).

During myocarditis, this finely tuned metabolic program is profoundly disrupted. A hallmark metabolic alteration is the shift from efficient aerobic oxidation via oxidative phosphorylation toward less efficient anaerobic glycolysis. This metabolic reprogramming arises as a consequence of mitochondrial damage and a hypoxic microenvironment and initially serves as an adaptive response to energy stress. However, it also represents a form of pathological compensation that further exacerbates cellular dysfunction and injury (51). The transition from oxidative phosphorylation to glycolysis directly precipitates an “energy crisis” within the heart, leading to contractile dysfunction, intracellular acidosis, enhanced oxidative stress, and cardiomyocyte death (52). As such, metabolic reprogramming constitutes a critical pathological nexus linking immune-mediated myocardial injury to the development of cardiac dysfunction and heart failure. Whether metabolic reprogramming can be reversed, and whether such reversal may attenuate inflammatory cardiomyocyte injury, has therefore emerged as a central focus of current research in myocarditis.

## 3 Glucose metabolism in the normal heart

The adult heart is a highly energy-dependent organ, and the maintenance of continuous, rhythmic contractile activity requires a stable and efficient supply of ATP (53). Under physiological conditions, cardiomyocytes predominantly rely on mitochondrial oxidative phosphorylation for energy production (54). Among available substrates, fatty acid β-oxidation represents the principal energy source, accounting for approximately 70%–90% of ATP generation, whereas glucose oxidation, lactate utilization, and ketone body oxidation serve as complementary contributors to cardiac energy metabolism (55). This fatty acid-dominant, multisubstrate metabolic configuration confers a high degree of metabolic flexibility, enabling the heart to maintain energy homeostasis under varying physiological conditions (56).

Although glucose is not the primary energy substrate in the normal heart, its metabolism plays an essential role in maintaining cellular homeostasis and supporting rapid responses to metabolic stress (57). Glucose uptake by cardiomyocytes is mainly mediated by members of the glucose transporter family, among which GLUT1 and GLUT4 are the most critical. GLUT1 is constitutively expressed on the cardiomyocyte plasma membrane and is responsible for basal glucose uptake, whereas GLUT4 is predominantly stored in intracellular vesicles and translocates to the plasma membrane in response to insulin stimulation or increased mechanical workload, thereby enhancing glucose uptake capacity (58). Under healthy conditions, this process is tightly regulated to prevent excessive reliance of cardiomyocytes on glucose as an energy source.

Once transported into cardiomyocytes, glucose is first phosphorylated by hexokinase to generate glucose-6-phosphate, which subsequently enters multiple metabolic pathways (59). Glycolysis constitutes the core pathway of glucose metabolism, and under physiological conditions, the pyruvate generated through glycolysis is primarily transported into mitochondria. There, it is converted into acetyl-CoA by the pyruvate dehydrogenase complex, enters the tricarboxylic acid cycle, and fuels oxidative phosphorylation. This metabolic routing ensures efficient utilization of the glucose carbon skeleton and maximizes ATP production (60).

In addition to glycolysis, a fraction of glucose-6-phosphate is diverted into the pentose phosphate pathway (PPP). In the normal heart, this pathway maintains a relatively low but stable level of activity (60). Its primary function is not direct energy production but the generation of nicotinamide adenine dinucleotide phosphate (NADPH), which is required to preserve intracellular redox balance and to provide substrates for nucleotide synthesis and reductive biosynthetic processes (61). The presence of the PPP enables cardiomyocytes to cope with oxidative stress under conditions of high metabolic demand while preventing excessive accumulation of metabolic byproducts.

The rate of glycolysis in cardiomyocytes is further constrained by regulatory mechanisms. Phosphofructokinase-2/fructose-2,6-bisphosphatase exhibits relatively limited activity in the heart and serves as an important brake on glycolytic flux. This feature helps prevent abnormal activation of glycolysis under non-pathological conditions and preserves the dominant role of fatty acid oxidation in cardiac energy metabolism (62). In parallel, NRF2 maintains low-to-moderate basal activity and supports antioxidant defenses by regulating the expression of key PPP enzymes, such as glucose-6-phosphate dehydrogenase and phosphogluconate dehydrogenase, thereby ensuring adequate NADPH supply (63).

Within this metabolic framework, glucose metabolism operates in close coordination with fatty acid oxidation, mitochondrial function, and redox regulation. By restricting excessive glycolytic flux and promoting the mitochondrial oxidation of pyruvate, cardiomyocytes achieve higher ATP production efficiency while avoiding lactate accumulation, intracellular acidification, and excessive generation of reactive oxygen species (64, 65). This metabolic state provides the foundation for long-term structural integrity and contractile function of the myocardium.

Taken together, glucose metabolism in the normal heart does not operate at high flux but rather functions as a tightly controlled, auxiliary metabolic pathway that cooperates with other energy substrates to maintain energy homeostasis (66). When inflammation, hypoxia, or mitochondrial dysfunction occurs, this finely balanced metabolic program is readily disrupted, which may represent one of the important metabolic prerequisites for the development of myocarditis.

## 4 Characteristics of glucose metabolic reprogramming in myocarditis

Metabolic reprogramming refers to the process by which cells undergo systematic adjustments of energy metabolic pathways in response to specific pathological or stress conditions, with the core feature being a redirection of substrate utilization and metabolic flux (67). In myocarditis, metabolic reprogramming is not an isolated metabolic event but rather an integrated pathological response driven by cardiomyocyte structural damage, mitochondrial dysfunction, and persistent activation of inflammatory signaling (68). A growing body of evidence indicates that cardiomyocytes in myocarditis undergo a metabolic transition from an efficient energy-producing state dominated by fatty acid oxidation and oxidative phosphorylation toward a glycolysis-enriched metabolic phenotype. This transition represents a critical initiating event in the metabolic abnormalities associated with myocarditis (69).

During the early stage of the disease, viral infection or immune-mediated injury can directly disrupt mitochondrial structure in cardiomyocytes, impair the integrity of the electron transport chain, and reduce the efficiency of oxidative phosphorylation (70). In the absence of a substantial reduction in ATP demand, cardiomyocytes compensate by upregulating glucose uptake and the expression of key glycolytic enzymes, thereby relying on glycolysis as an alternative energy source to maintain basic cellular functions. Experimental studies have demonstrated that CVB3 infection markedly induces the expression of hexokinase 2, phosphofructokinase, and pyruvate kinase M2, accompanied by a reduced proportion of pyruvate entering mitochondrial oxidation (71). Although enhanced glycolysis during this phase serves a short-term adaptive purpose, its ATP yield is considerably lower than that of oxidative phosphorylation.

As inflammation persists, metabolic reprogramming gradually shifts from a reversible compensatory response to a relatively stable pathological phenotype. Sustained elevation of glycolytic flux leads to lactate accumulation within myocardial tissue, and local microenvironmental acidification further suppresses mitochondrial function and weakens fatty acid oxidation capacity (71). Concurrently, a portion of glucose-derived metabolites is diverted into alternative metabolic pathways, including the pentose phosphate pathway and other biosynthetic branches. While these changes partially support antioxidant defenses and inflammatory cell functions, they also exacerbate metabolic imbalance and reduce overall energy efficiency (72). Persistent accumulation of lactate and reactive oxygen species not only directly injures cardiomyocytes but also acts as a metabolic signaling cue that promotes activation of pro-inflammatory pathways (73).

Metabolic reprogramming within immune cells further amplifies inflammatory responses in myocarditis. Monocytes, macrophages, and effector T cells infiltrating the heart exhibit a pronounced dependence on glycolysis under inflammatory conditions (74). Pro-inflammatory macrophages and Th1/Th17 cells require high levels of glycolytic activity to sustain cytokine production and effector functions, whereas anti-inflammatory macrophages and regulatory T cells preferentially rely on oxidative phosphorylation and fatty acid oxidation (75). In the myocardial inflammatory milieu, alterations in metabolic substrate availability and inflammatory signaling bias immune cell metabolism toward pro-inflammatory phenotypes, thereby hindering effective resolution of inflammation. In turn, metabolic reprogramming of immune cells exacerbates mitochondrial injury and metabolic dysfunction in cardiomyocytes through cytokine release, reactive oxygen species production, and accumulation of metabolic byproducts (76).

**Figure 1:**
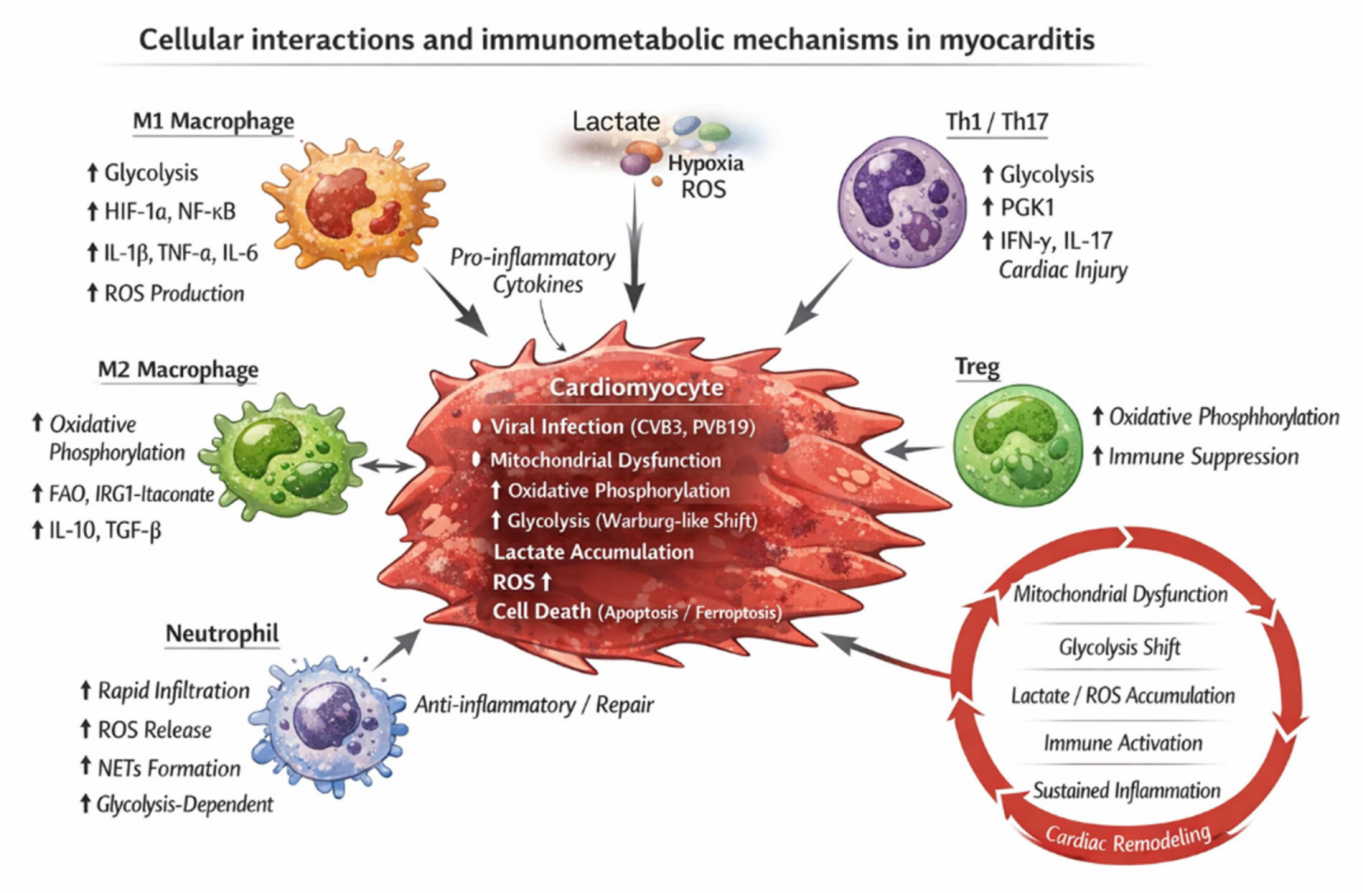
Cellular interactions and immunometabolic mechanisms in myocarditis. Viral infection (e.g., Coxsackievirus B3 and Parvovirus B19) or immune dysregulation directly injures cardiomyocytes, leading to mitochondrial dysfunction and a metabolic shift from oxidative phosphorylation toward glycolysis. Enhanced glycolysis is accompanied by lactate and reactive oxygen species (ROS) accumulation, promoting cardiomyocyte death, including apoptosis and ferroptosis, as well as the release of damage-associated molecular patterns (DAMPs). The inflammatory microenvironment subsequently recruits and activates immune cells. Pro-inflammatory macrophages (M1) and effector T cells (Th1/Th17) rely on glycolysis to sustain inflammatory functions and secrete pro-inflammatory cytokines, thereby exacerbating myocardial injury. In contrast, anti-inflammatory macrophages (M2) and regulatory T cells (Treg) preferentially utilize oxidative phosphorylation and fatty acid oxidation, contributing to inflammation resolution and tissue repair. Neutrophils rapidly infiltrate the heart during the acute phase and amplify inflammation through glycolysis-dependent ROS release and neutrophil extracellular trap formation. The reciprocal interaction between immune activation and metabolic reprogramming establishes a self-sustaining immunometabolic loop that ultimately drives myocardial fibrosis and adverse cardiac remodeling.

Through these interactions, a self-reinforcing positive feedback loop emerges between metabolic dysregulation and inflammation. Mitochondrial dysfunction promotes compensatory activation of glycolysis, while the accumulation of glycolytic intermediates and metabolites amplifies inflammatory signaling. Sustained inflammation further damages mitochondrial structure and function, thereby reinforcing metabolic abnormalities. This vicious cycle leads to the pathological fixation of metabolic reprogramming rather than a transient stress adaptation (Figure 1). Over time, cardiomyocyte metabolic flexibility declines markedly, and energy metabolism fails to recover to a normal state even after partial resolution of inflammation.

When the immunometabolic axis persists, myocarditis progresses into a chronic phase. Ongoing metabolic dysfunction and immune activation promote cardiomyocyte death, fibroblast activation, and extracellular matrix deposition, ultimately driving adverse cardiac remodeling. At this stage, metabolic reprogramming not only sustains myocardial injury but also provides a critical metabolic foundation for the progression of myocarditis toward dilated cardiomyopathy (77). Overall, glucose metabolic reprogramming in myocarditis can be conceptualized as a continuously evolving pathological cascade: Mitochondrial injury triggers compensatory glycolysis; metabolic byproducts and alternative pathways amplify inflammation; metabolic abnormalities become progressively fixed; and sustained metabolic–inflammatory interactions drive cardiac remodeling and eventual functional decompensation.

Although accumulating studies support the involvement of glucose metabolic reprogramming in myocarditis, several important limitations should be acknowledged. First, most mechanistic evidence is derived from viral myocarditis or experimental autoimmune myocarditis models, which capture specific inflammatory contexts but do not fully reflect the heterogeneity of human myocarditis. Differences in pathogenic triggers, immune landscapes, and disease kinetics among experimental models may partially explain inconsistencies in reported metabolic phenotypes. Second, current studies often infer metabolic states based on enzyme expression or pathway activation, whereas direct measurements of metabolic flux remain limited. This gap complicates interpretation of whether enhanced glycolysis represents a primary driver of myocardial injury or a secondary adaptive response to mitochondrial dysfunction. Finally, the relative contribution of cardiomyocyte versus immune cell metabolic reprogramming remains incompletely resolved. While both compartments exhibit glycolytic shifts, their temporal sequence and causal hierarchy during disease progression are not fully delineated, highlighting a key knowledge gap that warrants further investigation.

## 5 Regulatory mechanisms of glucose metabolic reprogramming

### 5.1 NRG1/ErbB signaling pathway and regulation of cardiac metabolic status

At the regulatory level, in models of cardiac injury and regeneration, activation of NRG1/ErbB2 signaling has been shown to induce a metabolic shift in cardiomyocytes from fatty acid oxidation toward glycolysis, providing readily available energy and biosynthetic precursors to support cell proliferation and tissue repair. This metabolic transition is considered adaptive in regenerative contexts. However, in inflammation-dominant pathological conditions such as myocarditis, aberrant activation of NRG1 signaling may exacerbate glycolytic dependence. When combined with inflammation-induced mitochondrial dysfunction, this shift may further compromise oxidative phosphorylation capacity. Although direct evidence linking NRG1 signaling to glucose metabolism regulation in myocarditis remains limited, its dual roles in metabolic modulation and immune regulation suggest that this pathway may contribute to the initiation and maintenance of metabolic reprogramming in myocarditis. (78).

### 5.2 NRF2-mediated regulation of the pentose phosphate pathway

Mechanistically, under conditions of inflammation and oxidative stress, NRF2-mediated activation of the PPP promotes NADPH production, thereby providing reducing equivalents necessary for glutathione regeneration and reactive oxygen species scavenging. In models of cardiac pressure overload and inflammation, NRF2 activation has been shown to alleviate oxidative injury and improve cardiac function. In the context of myocarditis, increased PPP flux may exert protective effects by limiting mitochondrial ROS accumulation. Conversely, excessive diversion of glucose-derived metabolites into the PPP may reduce substrate availability for mitochondrial oxidation and further enhance reliance on glycolysis. This dual role positions NRF2 as a critical regulatory node in glucose metabolic reprogramming in myocarditis, with its effects being highly context dependent (79).

### 5.3 Negative regulation of immunometabolism by the IRG1/itaconate axis

IRG1, also known as aconitate decarboxylase 1, and its metabolic product itaconate constitute a key regulator*y* axis in immune cell metabolic reprogramming. IRG1 is highly expressed in inflammation-activated macrophages and catalyzes the conversion of the tricarboxylic acid cycle intermediate cis-aconitate into itaconate. Itaconate is increasingly recognized as an endogenous immunometabolic regulator with both anti-inflammatory and antioxidant properties.

Itaconate suppresses inflammatory signaling by inhibiting succinate dehydrogenase activity, thereby limiting succinate accumulation and attenuating HIF-1α– and NF-κB-dependent pro-inflammatory pathways. In parallel, itaconate activates NRF2 signaling to enhance antioxidant defenses. In models of cardiac injury and inflammation, IRG1 deficiency has been associated with uncontrolled inflammatory responses and worsening cardiac function, whereas administration of exogenous itaconate derivatives, such as 4-octyl itaconate, significantly attenuates inflammation and tissue damage. In myocarditis, the IRG1/itaconate axis is considered an important endogenous negative feedback mechanism that counteracts pathological metabolic reprogramming by limiting excessive glycolytic dependence and pro-inflammatory polarization of immune cells (79).

### 5.4 PGK1-mediated enhancement of glycolysis and regulation of T-cell function

PGK1 is a key rate-limiting enzyme in glycolysis and also serves as an important molecular link between metabolic state and immune cell function. In experimental autoimmune myocarditis models, single-cell transcriptomic analyses have revealed significantly elevated PGK1 expression in cardiac-infiltrating CD4^+^ T cells, particularly Th17 cells. Upregulation of PGK1 is closely associated with enhanced glycolysis and suppression of oxidative phosphorylation (80).

Functional studies have demonstrated that PGK1 promotes Th17 cell differentiation and sustains their pro-inflammatory phenotype through regulation of the PDHK1–ROS axis. Pharmacological inhibition of PGK1 reprograms T-cell metabolism, reduces the proportion of Th1/Th17 cells, and increases regulatory T-cell populations, thereby markedly attenuating myocardial inflammation, fibrosis, and cardiac dysfunction. These findings indicate that PGK1 is not only a regulator of glycolytic flux but also a central hub linking glucose metabolic reprogramming to adaptive immune responses in myocarditis.

### 5.5 Other relevant pathways and summary

In addition to the pathways discussed earlier, the HIF-1α/mTOR signaling axis, STAT6 acetylation-dependent regulation, and the PI3K–AKT pathway have also been implicated in glucose metabolic reprogramming during myocarditis. These signaling networks may exert synergistic or antagonistic effects depending on cell type and disease stage, collectively shaping the metabolic–inflammatory microenvironment (81).

Overall, glucose metabolic reprogramming in myocarditis is not driven by a single molecule or pathway but rather results from the coordinated actions of multiple signaling networks operating in both cardiomyocytes and immune cells. NRG1 signaling influences cardiomyocyte metabolic status; the NRF2–PPP axis maintains redox balance; the IRG1/itaconate axis restrains excessive immunometabolic activation; and PGK1 plays a central role in T-cell metabolism and inflammatory amplification. Together, these mechanisms determine the magnitude, persistence, and pathological consequences of metabolic reprogramming and provide multiple potential entry points for targeted metabolic interventions (82).

## 6 Therapeutic advances targeting glucose metabolic reprogramming

Therapeutic strategies targeting glucose metabolic reprogramming are primarily centered on two objectives: reducing pathological dependence on glycolysis while restoring mitochondrial oxidative metabolism, and reprogramming immune cell metabolic states to limit pro-inflammatory effects and promote inflammation resolution. Current evidence is largely derived from animal models and *in vitro* systems, with most studies focusing on viral myocarditis and experimental autoimmune myocarditis (83).

Inhibition of glycolysis represents the most direct intervention strategy. 2-Deoxy-D-glucose (2-DG) reduces glycolytic flux by competitively inhibiting hexokinase-related steps. In CVB3-induced viral myocarditis models, enhanced glycolysis has been shown to facilitate viral replication and sustain inflammatory responses. Administration of 2-DG reduces viral titers and attenuates myocardial injury, suggesting that glycolytic inhibition within specific therapeutic windows may simultaneously interfere with pathogen replication and host inflammatory responses. However, a major limitation of this approach is that the target lies upstream in core energy metabolism and lacks cell-type specificity. Both cardiomyocytes and immune cells rely on glucose metabolism to maintain essential functions, and broad inhibition carries risks of insufficient energy supply, systemic metabolic disturbances, and off-target toxicity. These concerns are particularly relevant in fulminant myocarditis or in patients with limited cardiac functional reserve (84).

Activation of protective metabolic axes emphasizes the concept of “counteracting inflammation through metabolic homeostasis.” The IRG1/itaconate axis forms an endogenous negative feedback mechanism in inflammation-activated macrophages, with itaconate and its derivatives suppressing excessive inflammation while enhancing antioxidant responses. Previous studies have shown that IRG1 deficiency is associated with uncontrolled inflammation and deteriorated cardiac function, whereas exogenous itaconate derivatives, such as 4-octyl itaconate, effectively attenuate inflammation-related cardiac injury. A major limitation of this strategy is its strong context dependence. Differences in pathogen type, disease stage, and immune cell composition may influence the balance between viral control and inflammation suppression mediated by the itaconate axis, warranting more systematic validation (85).

Combined antioxidant and metabolic remodeling strategies have also gained increasing attention. α-Lipoic acid has been reported to suppress inflammation and improve cardiac function in CVB3-induced myocarditis models through mechanisms associated with metabolic reprogramming. NRF2-related strategies enhance pentose phosphate pathway flux and NADPH availability, thereby improving redox homeostasis. These approaches have demonstrated clear cardioprotective effects in models of cardiac stress and are considered potentially beneficial in inflammatory cardiac injury. However, such strategies may exert bidirectional effects on glucose metabolism: While enhancing antioxidant capacity, they may also promote diversion of substrates toward non-mitochondrial oxidative pathways under certain conditions. Consequently, the corrective effects of metabolic reprogramming may depend on disease stage and cell-type composition (86).

Overall, current therapeutic approaches face three shared challenges. First, substantial heterogeneity exists between experimental myocarditis models and human disease, including differences in pathogenic agents, immune landscapes, and metabolic backgrounds, resulting in variable responses to the same metabolic intervention. Second, metabolic interventions exhibit strong time dependence. Premature inhibition of glycolysis during the acute phase may impair essential antiviral immunity or stress-induced energy supply, whereas later stages require suppression of pathological fixation and restoration of metabolic flexibility. Third, many metabolic targets are involved in fundamental cellular processes, and the lack of tissue and cell-type specificity represents a major bottleneck for clinical translation. Therefore, dosing regimens, delivery routes, and combination strategies must be redesigned with safety as a central consideration. Despite encouraging results from preclinical studies, the clinical translation of metabolic interventions in myocarditis faces substantial challenges. Metabolic dependencies vary across disease stages, with early phases requiring intact antiviral and stress–response metabolism, whereas later stages may benefit from suppression of pathological metabolic fixation.

Safety concerns represent a major barrier. Broad inhibition of glycolysis may compromise myocardial energy supply or impair protective immune responses, particularly in fulminant myocarditis or patients with limited cardiac reserve. Furthermore, metabolic heterogeneity among patients, influenced by age, metabolic comorbidities, and systemic inflammatory status, complicates patient selection and therapeutic timing.

These considerations underscore the need for stage-adapted, cell-type–aware metabolic interventions before clinical application can be realistically achieved.

## 7 Discussion

**Figure 2:**
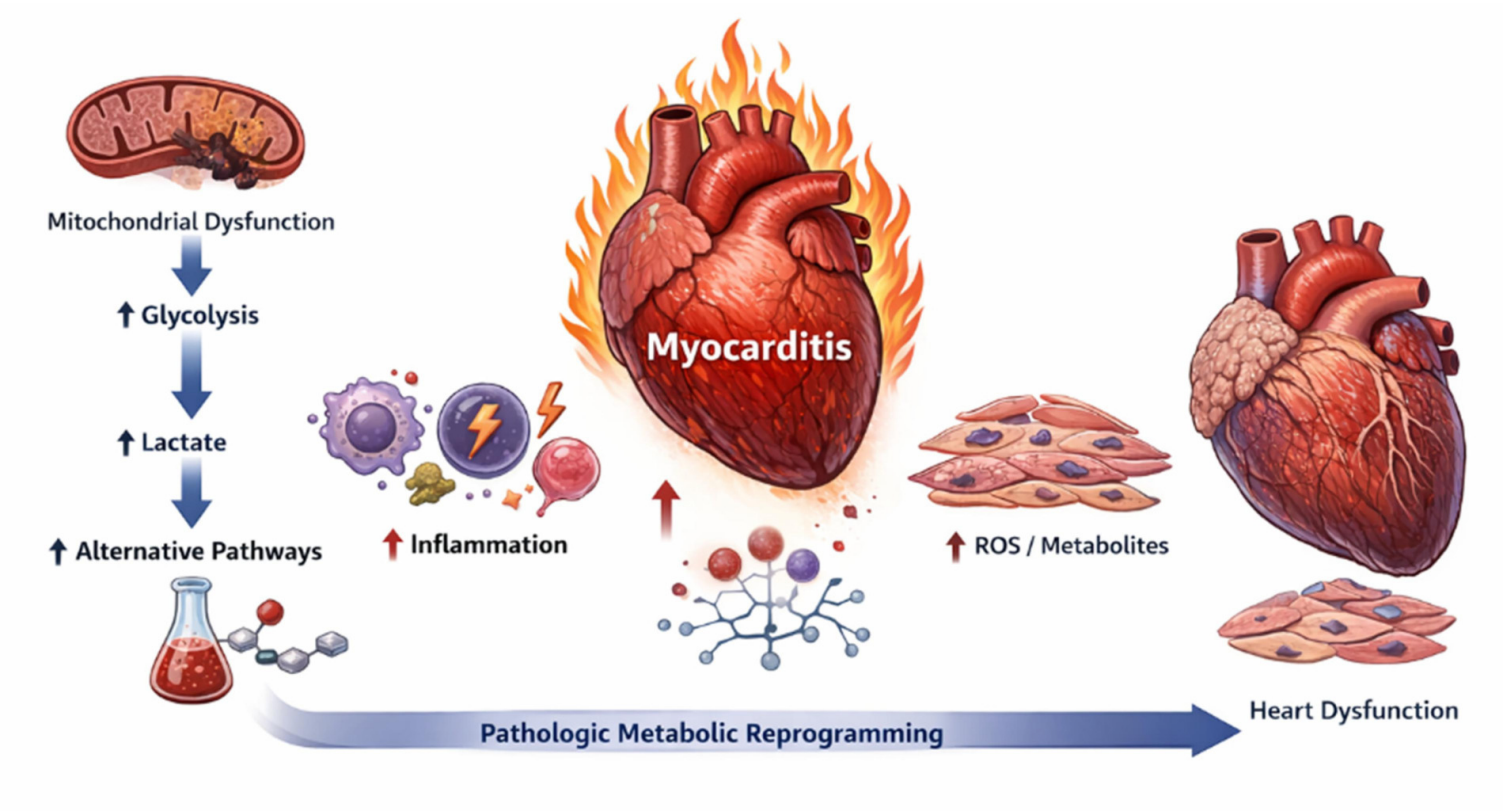
Schematic overview of glucose metabolic reprogramming in myocarditis. Viral infection or immune dysregulation induces mitochondrial dysfunction in cardiomyocytes, leading to a metabolic shift from oxidative phosphorylation toward glycolysis. Enhanced glycolysis is accompanied by lactate accumulation and activation of alternative metabolic pathways, resulting in increased reactive oxygen species production and amplification of inflammatory responses. The reciprocal interaction between metabolic abnormalities and immune cell activation establishes a self-sustaining immunometabolic feedback loop, which becomes pathologically fixed over time and ultimately drives myocardial injury, fibrosis, and cardiac dysfunction.

Despite the promising potential of therapeutic strategies targeting glucose metabolic reprogramming, research on myocarditis continues to face several important challenges. Current limitations include the lack of highly specific animal models that accurately recapitulate the heterogeneity of human myocarditis, as well as substantial barriers to clinical translation. In particular, the limited specificity of metabolic interventions raises concerns regarding unintended interference with normal cardiac energy supply. In addition, pronounced interindividual variability, influenced by external factors such as age, hyperglycemia, and gut microbiota composition, contributes to heterogeneous therapeutic responses. To improve conceptual integration, key metabolic regulators, affected cell types, and therapeutic implications are summarized in schematic form (Figure 2) and comparative frameworks, facilitating a systems-level understanding of immunometabolic regulation in myocarditis.

Potential risks associated with metabolic interventions should not be underestimated. Sustained inhibition of glycolysis may lead to insufficient energy production or exacerbation of acidosis, while certain agents, such as 2-DG (84), may induce oxidative stress or affect non-target cells at high doses. Moreover, combining metabolic modulation with immunosuppressive strategies may increase susceptibility to infection, which is of particular concern in viral myocarditis, where adequate antiviral immunity is essential.

Future research directions should focus on several key areas. Single-cell multiomics analyses will be instrumental in elucidating cell type-specific mechanisms of metabolic reprogramming. Integrated multiomics approaches may further uncover complex metabolic–inflammatory interaction networks underlying disease progression. In parallel, personalized therapeutic strategies based on individual metabolic profiles, such as hyperglycemic status or age-related metabolic alterations, may help optimize treatment efficacy. The development of novel therapeutic targets, including the IRG1/itaconate pathway or selective activation of pentose phosphate pathway branches, also warrants further investigation. Importantly, interdisciplinary collaboration integrating metabolomics, immunology, and cardiology will be essential to accelerate the translation of basic mechanistic insights into clinically applicable, precision-based interventions (87).

## 8 Conclusion

This review highlights glucose metabolic reprogramming as a central pathological process linking immune activation to myocardial injury in myocarditis. Rather than representing a uniform metabolic shift, glucose reprogramming emerges as a dynamic and context-dependent response shaped by disease stage, cell type, and inflammatory burden.

A major challenge moving forward lies in distinguishing adaptive metabolic responses from maladaptive fixation, as well as identifying patient subsets most likely to benefit from metabolic intervention. Current therapeutic strategies remain constrained by limited cell specificity, timing sensitivity, and safety concerns, underscoring the need for precision-guided approaches.

Future studies integrating single-cell and spatial multiomics with functional metabolic assays will be essential to resolve these uncertainties and to translate immunometabolic insights into clinically viable interventions.
